# Exploring Networks of Lexical Variation in Russian Sign Language

**DOI:** 10.3389/fpsyg.2021.740734

**Published:** 2022-01-05

**Authors:** Vadim Kimmelman, Anna Komarova, Lyudmila Luchkova, Valeria Vinogradova, Oksana Alekseeva

**Affiliations:** ^1^Department of Linguistic, Literary, and Aesthetic Studies, University of Bergen, Bergen, Norway; ^2^Galina Zaitseva Centre for Deaf Studies and Sign Language, Moscow, Russia; ^3^Department of English Stylistics, English Language Faculty, Moscow State Linguistic University, Moscow, Russia; ^4^Inclusive Programs Department, Garage Museum of Contemporary Art, Moscow, Russia; ^5^School of Psychology, University of East Anglia, Norwich, United Kingdom

**Keywords:** lexical variation, phonological variation, Russian Sign Language, lexical database, graph theory

## Abstract

When describing variation at the lexical level in sign languages, researchers often distinguish between phonological and lexical variants, using the following principle: if two signs differ in only one of the major phonological components (handshape, orientation, movement, location), then they are considered phonological variants, otherwise they are considered separate lexemes. We demonstrate that this principle leads to contradictions in some simple and more complex cases of variation. We argue that it is useful to visualize the relations between variants as graphs, and we describe possible networks of variants that can arise using this visualization tool. We further demonstrate that these scenarios in fact arise in the case of variation in color terms and kinship terms in Russian Sign Language (RSL), using a newly created database of lexical variation in RSL. We show that it is possible to develop a set of formal rules that can help distinguish phonological and lexical variation also in the problematic scenarios. However, we argue that it might be a mistake to dismiss the actual patterns of variant relations in order to arrive at the binary lexical vs. phonological variant opposition.

## Introduction

Sign languages, like all natural languages, are variable, with variation present at the phonological, lexical, and grammatical levels. The choice of variant can depend on the region of the signer, their age, and other sociolinguistic factors, including ones specifically relevant to sign languages, such as the type of school, and the presence of signing family members (Sutton-Spence et al., 1990; Schermer, 2004; Schembri and Johnston, 2012; Stamp et al., 2015; Palfreyman, 2019; Chen and Gong, 2020). Many studies focus on investigating these factors that explain the choice of variant. However, before exploring these factors, researchers need to conduct a more technical step of defining what constitutes different variants, and determining which level of variation is concerned. In this paper, we specifically discuss the problem of distinguishing phonological and lexical variants of signs.

### The Puzzle of Lexical and Phonological Variation

When studying variation in signs of sign languages, researchers usually distinguish between lexical variants and phonological variants (Johnston and Schembri, 1999; Lucas et al., 2001; Schermer, 2004; Stamp et al., 2014; Fenlon et al., 2015; Chen and Gong, 2020). The two cases that need distinguishing are:

1. For concept X, there are two signs 1 and 2 that are formally related. 1 and 2 thus represent a single lexeme with different phonological realizations.
2. For concept X, there are two signs 1 and 2 that are distinct in their shape (unrelated). 1 and 2 are thus separate lexemes that are variant expressions of a single meaning.^1^

**FIGURE 1:**
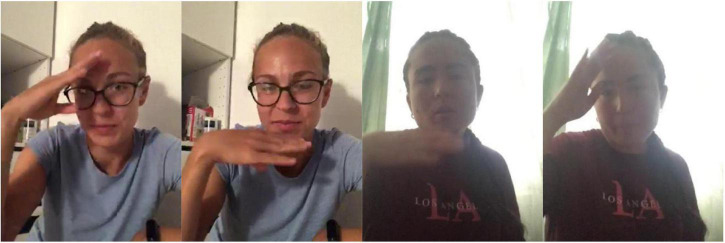
FATHER-1 (two frames, watch here: https://osf.io/u2nej/) and FATHER-2 (two frames, watch here: https://osf.io/wt8dh/).

Consider the following simple example. In Russian Sign Language (RSL), the concept FATHER can be expressed by the following signs (Figure 1). The signs come from the lexical database of variation in RSL, which will be introduced in detail in Section “The database of lexical variation in RSL” below. The two signs are clearly formally related: they share the handshape, the orientation, the locations, and the type of movement; only the direction of movement is different between the two signs: in FATHER-1 the hand moves from the forehead to the chin, and in FATHER-2 from the chin to the forehead.

**FIGURE 2:**
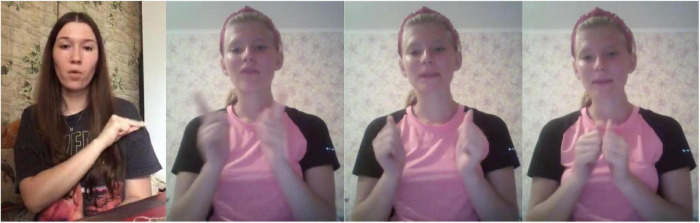
EDUCATOR-1 (1 frame, watch here: https://osf.io/8y2dn/) and EDUCATOR-2 (3 frames, watch here: https://osf.io/5aygc/).

Compare this to the following two RSL signs, expressing the concept of EDUCATOR (the sign for the person working typically at a boarding school for deaf children who is more responsible for the discipline than for education), Figure 2. These two signs have no formal overlap (and the second one is probably a compound), so it is logical to treat them as completely separate lexical items (lexemes).

The criterion for distinguishing lexical variants from phonological variants that is most often used in published research and in existing dictionaries and lexical databases of sign languages is the following*:*

(1) If two signs for the same concept differ in one major phonological parameter (handshape, orientation, movement, location), then they are phonological variants of the same lexeme. Otherwise, they belong to separate lexemes.

Several additional notes are in order. First, if two signs for the same concept use different iconic bases (informally, they draw a different picture), they are considered separate lexemes, even if they only differ in a single phonological parameter (Lucas et al., 2001; König et al., 2008). Second, handedness (one vs. two-handed realization) is often not considered a distinguishing feature because both adding and removing the second hand is a very common phonetic/phonological process in sign languages (Johnston and Schembri, 1999; Fenlon et al., 2015). Third, whether mouthing is used to distinguish lexemes is questionable because the status of mouthing itself is contested (see a discussion in Fenlon et al., 2015). These additional considerations are important issues, but we do not discuss them in this paper.

One obvious drawback of this criterion is that it is quite arbitrary. Why would signs sharing three out of the four, but not two out of the four parameters considered related?^2^ Furthermore, the notion of the major phonological parameter itself is theoretically questionable, as many theories of sign language phonology argue for a different (hierarchical) representation of phonological structure of signs (see Brentari, 2012; van der Hulst and van der Kooij, 2021 for an overview).

Another problem concerns relying on these phonological (also known as sublexical) parameters in sign languages, as discussed by Mudd et al. (2020) and Lutzenberger et al. (2021): in order to make a judgment whether, e.g., movement in two signs is the same or different, it is necessary to know which movement differences are phonological, and which are phonetic in the specific sign language. For most sign languages, phonological inventory has not been described in enough detail, and for some sign languages it has been claimed that phonology and thus phonological categories are only emerging (Israel and Sandler, 2011).

However, even if we accept the validity of the criterion, and settle on a common solution for the additional complications mentioned above, when we analyze the actual possible relations between multiple variant signs used to express the same concept, we are faced with contradictions. These contradictions will be the focus of this paper, and the possible scenarios that lead to contradictions are discussed in detail in Section “Problematic scenarios”.

However, as a preview, consider the following hypothetical scenario, which we call the Chain Scenario. Imagine that concept X can be expressed by signs 1, 2, and 3. Signs 1 and 2 are identical but for the handshape. Signs 2 and 3 are identical but for the movement. This means that by the criterion (1) above, signs 1 and 2 are one lexeme (phonological variants of the same sign), and signs 2 and 3 are one lexeme, but signs 1 and 3 are not the same lexeme. This is a contradiction. As we will show throughout the paper, this is not a hypothetical only scenario, but a very common occurrence, at least in RSL. Furthermore, this is the simplest of the complex scenarios that are found in the lexical variation database of RSL.

To the best of our knowledge, this issue of distinguishing phonological vs. lexical variants in the complicated scenarios we describe in this paper has not been analyzed in depth in any previous research. In most papers on lexical variation in sign languages, the focus is on connecting the choice of variant to sociolinguistic factors (e.g., Stamp et al., 2015; Chen and Gong, 2020; Mudd et al., 2020). The authors typically use the criterion in (1) to isolate separate lexical variants of signs, and then explain the distribution of these lexical variants. In some studies, the focus is on measuring variability quantitatively (e.g., Israel and Sandler, 2011; Lutzenberger et al., 2021), where both lexical and phonological variability is taken into account in order to calculate a variability metric, but the focus is again not on distinguishing lexical vs. phonological variants, and the cases relevant to our paper are not analyzed.

### Why Distinguish Lexical and Phonological Variation

It is prudent to ask why it is necessary to distinguish lexical and phonological variation at all. In principle, it is possible to solve the puzzle outlined above as well as further problems by simply abandoning the distinction, and treating all minimally formally distinct expressions of the same concept equally. However, there are some arguments in favor of trying to salvage this distinction.

First, for the purposes of lexicography it is necessary to distinguish lexemes from phonological variants, because dictionaries are typically organized around lexemes, and the phonological variants are discussed within entries devoted to specific lexemes (Johnston and Schembri, 1999; Kristoffersen and Troelsgård, 2010; Fenlon et al., 2015; Hochgesang et al., 2018).

Second, it is plausible to hypothesize that two lexemes for the same concept and two phonological variants of the same lexeme would be represented differently in the mental lexicon. While some might question the psychological reality of this difference, it is an empirical question whether these categories are psychologically real, and before we investigate it experimentally, we need to descriptively settle on the boundaries of these categories.

Finally, the distinction can also be relevant for other linguistic questions. For example, in our preliminary research on RSL we discovered that signs from different semantic fields are different with respect to the type of variation. Specifically, kinship terms and color terms in RSL typically have a large amount of phonological variation, while lexical variation is lower than for signs related to school and education. There is an intuitive explanation for this pattern: school-related signs are developed in specific deaf schools (Schembri and Johnston, 2012), and thus completely different unrelated variants emerge and are preserved, while color terms and kinship terms are less school-dependent, and thus the different variants either have a common source or interact and converge more easily. However, it is not possible to even describe this pattern if we abandon the two categories of variation.

For these reasons we consider it valuable to discuss the distinction further using novel data from RSL. Our final argument, however, will be in favor of acknowledging that the possible relations between variants go beyond the binary distinction of lexical vs. phonological.

## Materials and Methods

### The Database of Lexical Variation in Russian Sign Language

The current paper is based on the initial stages of the analysis of a database of lexical variation in RSL^3^ : https://rsl-research-explore.garagemca.org/. It was created by the Garage Museum of Contemporary Art in Moscow, with participation of sign language linguists. The database was collected using a website where participants were asked to record themselves signing isolated signs from several semantic fields^4^. The participants were specifically instructed to record multiple variants if they could recall them, starting with the one they themselves used most frequently. Data collection took place in the summer of 2020. Participation was on a purely voluntary basis.

The concepts selected for the questionnaire came from the following semantic fields: kinship terms, color terms, school-related lexicon, numerals. Kinship terms and color terms have been widely investigated for other sign languages, including investigations of lexical variation for these fields (Lucas et al., 2001; Schembri and Johnston, 2012; Stamp et al., 2014; Mudd et al., 2020). School-related lexicon was chosen because we assumed that such concepts would indeed vary considerably between different regions due to the important role deaf schools play in sign language emergence and transmission (Schembri and Johnston, 2012). Finally, anecdotal reports said that numerals in RSL do not vary across different regions, so we wanted to empirically test these reports. The total number of concepts included was around 90, excluding the numerals. We expected that filling the questionnaire would take 20-30 min.

The explanation of the purpose of the research, the instructions, and the questions in the sociolinguistic questionnaire (see below) were presented in both RSL and Russian. However, the stimuli in the questionnaire were words in written Russian. While it is well known that using written language as stimulus is not optimal (van Herreweghe and Vermeerbergen, 2012), this method is unavoidable in a large-scale online data collection study of lexical variation. We could not use video recordings of the signs as stimuli as this would obviously influence the participants, and many of the concepts we were interested in are not easily representable by pictures. Since we only collected isolated lexemes, we consider direct influence of written language to be restricted to mouthing. However, for this reason, this database cannot be used to analyze mouthing accompanying the signs.

In addition to collecting the recording of the signs, we collected socio-linguistic data about the participants, namely their dates of birth, gender, place of birth and places where they lived for a considerable period of time, age of acquisition of RSL, and deaf and hard-of-hearing relatives.

While more than 600 people started filling out the sociolinguistic questionnaire, 279 recorded two or more signs (it was possible to stop recording at any moment in the questionnaire). More than 19 000 videos (one video per concept per participant) were recorded. Due to the on-line format of the questionnaire, the participants do not constitute a representative sample of the population: the majority of them were in the 18–35 age range, and almost half of the signers coming from Moscow. Nevertheless, the database contains a large amount of variation that needs future linguistic and sociolinguistic analysis.

In this paper, we do not describe or analyze sociolinguistic factors that can explain variation and focus on the specific task of the linguistic analysis of variation in terms of the phonological vs. lexical opposition. This will thus serve as the basis for further annotation of the database, and the necessary first step for future sociolinguistic analysis. Furthermore, the majority of data analyzed so far concerns color terms and a few kinship terms. The video recording of all the signs discussed in this paper can be found here: https://osf.io/7h3f6/.

### Data Annotation

We annotated the data manually by describing each variant sign for a concept and assigning it a label (e.g., FATHER-1, FATHER-2, etc. assigned in order of occurrence in the database). In determining what constitutes separate variants, we used the following principles.

First, if one signer produced two signs in one video, these two signs are clearly perceived as separate variants by the signer, and we annotate them as such. This was especially useful for some controversial cases. For example, it is possible to analyze the variants of FATHER in Figure 1 not as two separately represented variants, but as a result of applying metathesis based on context^5^. However, such variants were in fact produced together by the same signers, and thus they are analyzed as distinct variants.

Second, we considered variants distinct if they were clearly different in at least one of the major parameters (handshape, orientation, location, movements). For the reasons discussed in Section ‘‘The database of lexical variation in RSL,’’ mouthing was not analyzed at all. Finally, we acknowledge that the decision of what constitutes the same or different major parameters (e.g., the same or different handshapes) in two variants is a subjective judgment. Our annotation was guided by our knowledge of the phonology of RSL based on many years using, studying and researching RSL,^6^ but not on a published formal inventory of phonological units, as such an inventory for RSL does not exist at the moment. As discussed above, this is a common methodological concern for studies of variation for most sign languages (Mudd et al., 2020).

For this study, we do not consider fingerspelling (although fingerspelling should probably be analyzed as separate lexemes), and we do not consider compounds. Compounds sometimes have parts which also serve to express the same meaning as a single sign and thus also complicate the system considerably. We leave this issue for future research.

Note that even if some of our annotations turn out to be erroneous (e.g., two variants should in fact be analyzed as a single variant, or vice versa), this can only invalidate some of the specific examples in the rest of the paper, but not the theoretical arguments about possible variant networks.

### Graph Theory

In the remainder of the paper, we will discuss the hypothetical and actual networks of variants. We argue that it is useful and insightful to represent these networks graphically, as such presentation makes the relations we are interested in intuitively clear. In order to do so, we need to use some basic notions from graph theory (Wilson, 1996). We introduce them here, and explain how they relate to the possible relations between variants of signs expressing a concept.

Graphs consist of vertices and edges that connect pairs of vertices. In our case, variants are vertices, and edges represent the relation of phonological relatedness between vertices/variants. Two vertices that are connected by an edge are called adjacent vertices. In our case, this means that two phonologically related variants [by (1)] are always adjacent vertices in the graph representation^7^.

**FIGURE 3:**
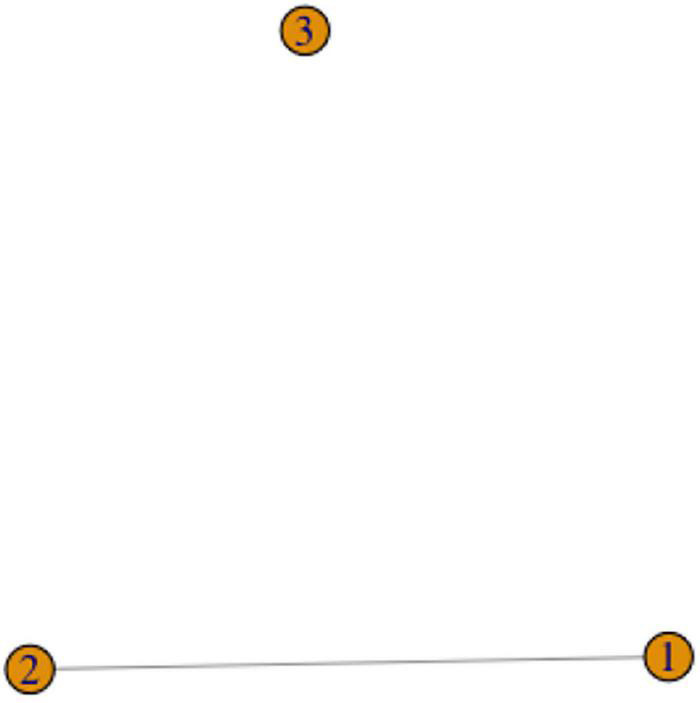
A graph with two components.

For example, recall the signs for FATHER in Figure 1. These two variants are phonologically related, and thus would be represented as two vertices connected by an edge in a graph representation (for example, as vertices 1 and 2 in Figure 3 below).

Graphs might have components, where components are connected parts of the graph that are not connected to each other. For instance, the graph in Figure 3 has two components: (1,2) and 3. In the case of graphs for sign variants, it is clear that separate components must belong to separate lexemes. However, the difference between separate lexemes and phonological variants is not reducible to the difference between components and vertices within a component, as the Chain Scenario discussed above shows. We will thus focus on exploring possible configurations within components.

A cycle is defined as a part of the graph that can be represented as a sequence of edges that are all distinct and that join a sequence of vertices such that this sequence starts and ends in the same vertex while no other vertices are repeated. In Section “Problematic scenarios” we show why this notion is relevant to analysis of sign variants (see also the figures there).

Graph theory has been used in linguistics in several domains. The most common applications of graph theory are to analyze semantic (Sigman and Cecchi, 2002, review in Borge-Holthoefer and Arenas, 2010), phonological (Vitevitch, 2008), and orthographic (Trautwein and Schroeder, 2018) lexical networks. The main approach here is to construct a large network representing a considerable part of a lexicon in a language, and then describe these networks in quantitative terms from graph theory, such as the average path length and clustering coefficient. The actual network under study is compared against a random network of the same size, in order to assess whether, e.g., the aforementioned average path length and clusterting coefficient are non-random. For instance, it has been shown that such lexical networks possess small-world characteristics, where the average path length is comparable to random networks, but the cluster coefficient is much higher. These properties of lexical networks seem to correlate with psycholinguistic evidence, e.g., on the role of phonological neighborhood density (Vitevitch, 2008). A study that applied a community detection technique, the Louvain method in particular, to language data (Siew, 2013) used a giant component of 6,508 words from the phonological network used in the Vitevitch (2008) study to identify communities within the network and then compare lexical and phonological characteristics of words in these communities. The Louvain method, commonly used in such studies, is tailored specifically for large networks (Blondel et al., 2008).

Another domain of application of graph theory is in computational linguistics, where different types of graph representations have been used to represent linguistic data and to perform various NLP tasks (see Kuhlmann and Oepen, 2016 for an overview of linguistic resources using graph representations). This domain is also closely connected to another related field of algorithmic community detection (Fortunato, 2010). Community detection algorithms are usually applied in cases of very large networks in order to discover the underlying structure of the network (Girvan and Newman, 2002; Fortunato, 2010). One example of applying community detection is represented by Jurgens (2011), where this technique was used to induce word senses from corpus data by detecting communities (interconnected parts of graphs) in a word co-occurrence graph.

One study that is in spirit similar to ours in that it focuses on relatively small graphs, albeit in a completely different linguistic domain, is Piperski(’s (2014) proposal to use graph theory to analyze linguistic complexity. In this study, the author proposed to measure linguistic complexity (in terms of form-meaning mappings) by applying some measures from graph theory.

Concerning lexical variation in sign languages, Chen and Gong (2020) used clustering, which is a statistical technique related to graph theory, to detect dialects in lexical signs in Chinese Sign Language. However, they only looked at what they considered to be lexical variants, for which they used the same criteria as elsewhere in the literature, and did not explore the question of the boundary between lexical and phonological variation. Similarly, Mudd et al. (2020) calculated lexical distance between Kata Kolok signers using lexical variation as basis for calculation; this also creates an underlying graph representation of signers in the community. However, they also did not analyze sign variant networks, as this was not part of their research question.

To sum up, while graph theory and community detection has been used in various domain of linguistics, it has not been applied to the phenomenon that we consider in this paper, namely analyzing variant networks to distinguish lexical and phonological variants. Given the relatively small sizes (in terms of the numbers of vertices and edges) of the networks considered here, we focus not on quantifying various measures over these graphs (such as average path length or clustering coefficient) or detecting communities algorithmically, but on describing the specific configurations that we find in the data in relation to the question of distinguishing lexical and phonological variants.

## Problematic Scenarios

In this section, we explore five scenarios where some variants are phonologically related to some other variants, which leads to contradictions or at least difficulties in distinguishing phonological and lexical variants. These scenarios are the Chain Scenario, the Cycle Scenario, the Overlapping Cycles Scenario, the Shared Vertex Scenario, and the Connected Component Scenario.

The list is not exhaustive: it is based on examples we found in the data. Thus, for each of these scenarios, we give actual RSL examples from the database of lexical variation. Afterward, we discuss some examples of complete variant networks for color terms demonstrating that, in actuality, multiple problematic scenarios can concern even a single concept.

### The Chain Scenario

**FIGURE 4:**
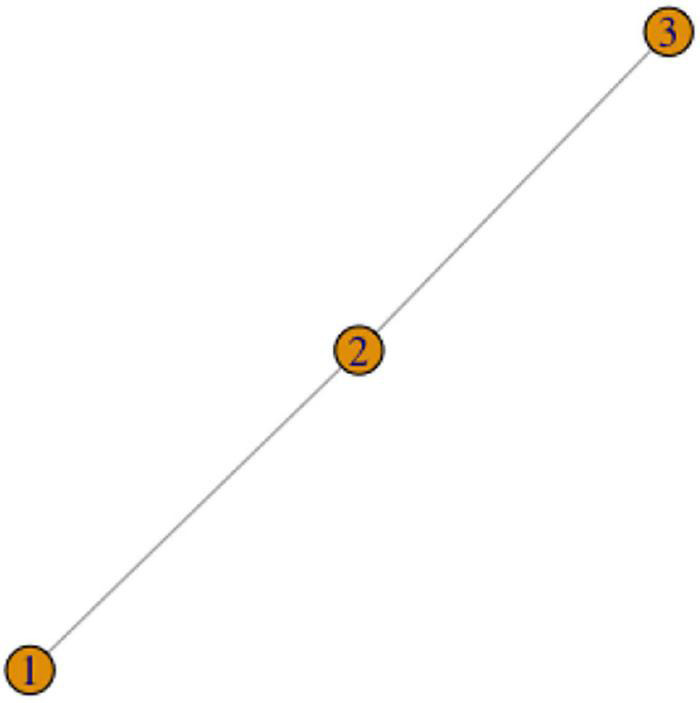
The Chain Scenario.

We introduced the Chain Scenario in Section “The puzzle of lexical and phonological variation,” but we repeat the description here. Imagine that concept X can be expressed by signs 1, 2, and 3. Signs 1 and 2 are identical but for the handshape. Signs 2 and 3 are identical but for the movement. This means that by the criterion (1), signs 1 and 2 are one lexeme (phonological variants of the same sign), and signs 2 and 3 are one lexeme, but signs 1 and 3 are not the same lexeme. This situation can also be represented as a graph in Figure 4.

**FIGURE 5:**
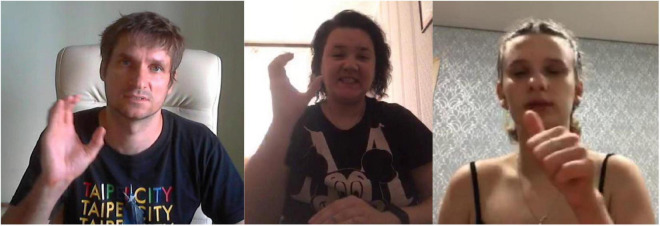
DARK.BLUE-11 (repeated downward movement not depicted), DARK.BLUE-12 (repeated wrist rotation not depicted), DARK.BLUE-13 (repeated wrist rotation not depicted). Watch the video recordings here: https://osf.io/7h3f6/.

This scenario is a very common phenomenon in the RSL database. Consider the following example. The concept DARK.BLUE in RSL has many variants (as we will further discuss in Section “The Connected Component Scenario”), but we will focus on three of them in this section, Figure 5. The first variant has the C handshape, making small repeated downward movements in the neutral space. The second variant has the same handshape and location, but the movement is the rotation of the wrist. The third variant has the A handshape, and the same movement and location as in the second variant.

The three variants thus exactly exemplify the Chain Scenario from: the first and second variants are only distinguished by one parameter (the movement), and the second and third are distinguished by one parameter (the handshape), but if we compare the first to the third, we observe two major parameter differences. How are we to analyze these variants in terms of the number of lexemes, and which variant should belong to which lexeme? We offer a solution in Section “A possible system of rules.”

### The Cycle Scenario

**FIGURE 6:**
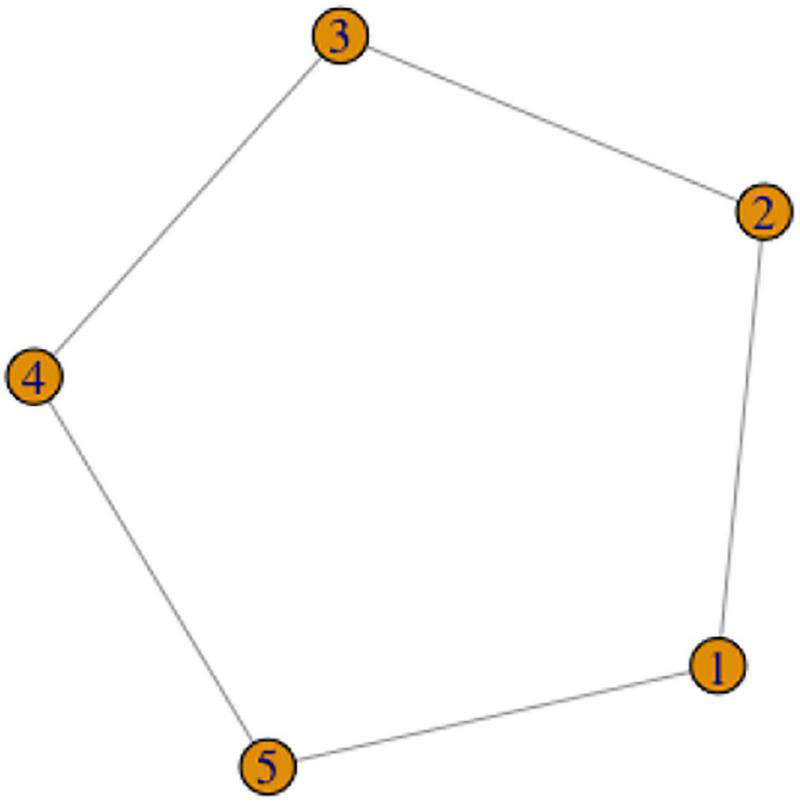
The Cycle Scenario.

In section “Graph theory,” we defined cycles in graph theory. A cycle is a sequence of edges that are all distinct and that join a sequence of vertices such that this sequence starts and ends in the same vertex while no other vertices are repeated. A simple example is that a concept X is expressed by variants 1, 2, 3, 4 and 5, such as the following pairs of variants can be defined as being phonologically related: (1,2), (2,3), (3,4), (4,5), (5,1) (Figure 6).

**FIGURE 7:**
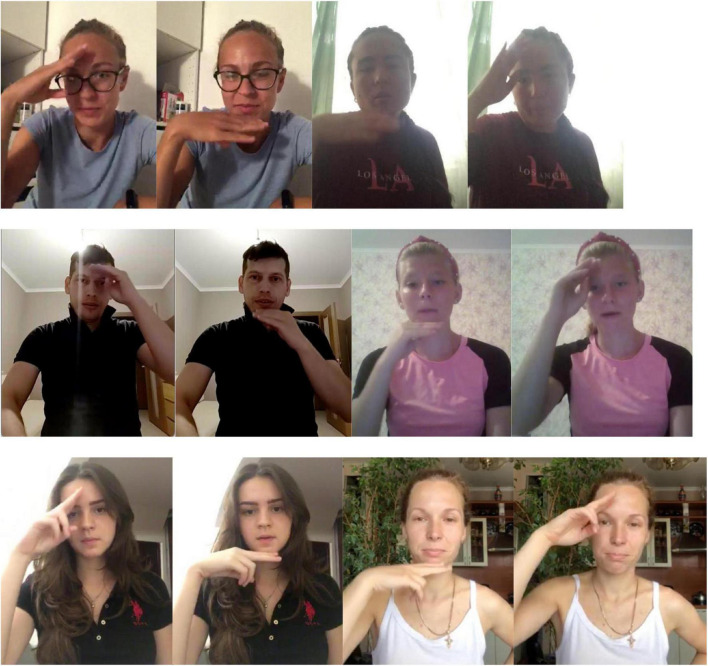
The six variants of FATHER (two frames each; in signs 1, 3 and 5, the hand moves from the forehead to the chin, in signs 2, 4 and 6, the hand moves from the chin to the forehead; watch the video recordings here: https://osf.io/7h3f6/).

This scenario is also a common occurrence in the database, and is best illustrated by the variants for the concept “father” (see also the color “white” in Supplementary Materials). This concept has six distinct realizations in RSL (Figure 7). Variants 1 and 2, variants 3 and 4, and variants 5 and 6 are all distinguished by the same component, namely variants 1, 2, and 3 have the movement from the forehead to the chin, and variants 4, 5, and 6 have the movement from the chin to the forehead. At the same time, variants 1 and 2 share the handshape (flat hand), and so do variants 3 and 4 (palm bent), and variants 5 and 6 (the H handshape).

How are we to analyze this system in terms of lexemes? First, variants 1 and 2 should belong to one lexeme, variants 3 and 4 should belong to one lexeme, and variants 4 and 5 should belong to one lexeme. The variants in these pairs are distinguished by movement direction only. Second, variants 1, 3, and 5 should belong to one lexeme, and variants 2, 4, and 6 should belong to one lexeme. The variants in these triplets are distinguished by handshape only. However, also by the same logic, variants 1 and 4, 1 and 6, 2 and 3, and 2 and 5 should not belong to the same lexemes, because they are different with respect to both movement and handshape.

**FIGURE 8:**
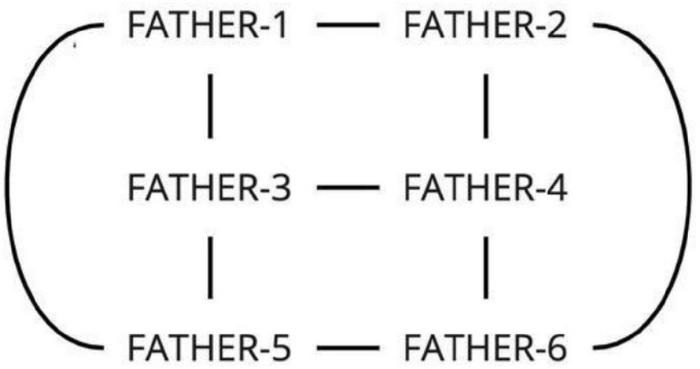
A graph representation for the variants of FATHER.

The relations between the variants are graphically represented in Figure 8.

The Cycle Scenario is intuitively different from the Chain Scenario because of the high degree of interconnectedness of the variants. While in a simple chain two variants on the ends of the chain are only connected to one other variant, in a cycle each variant is connected to at least two other variants. Intuitively, then, it becomes difficult to separate any of the variants into one lexeme without the rest of the variants that are so tightly connected to them.

### The Overlapping Cycles Scenario

**FIGURE 9:**
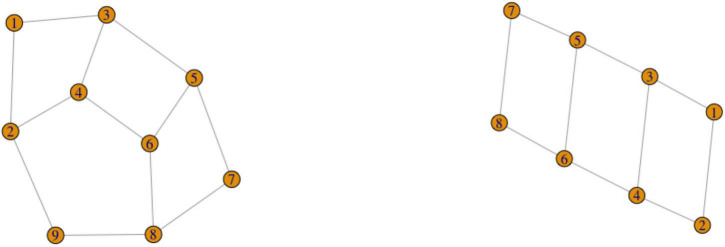
The Overlapping Cycles Scenario (left). A cycle left after removing one vertex (right).

Sometimes a network of vertices is interconnected, but does not form a single cycle, as there is no path through all the vertices such that the edges are distinct, and each vertex except for the first one is repeated. One such case is represented in Figure 9, left. Note that if one vertex is removed (vertex 9), the remaining graph is a cycle, as in Figure 9, right.

Thus, technically this is a different scenario from the Cycle Scenario above. However, in this scenario each vertex is still connected to at least two other variants within the component, and as such, we might want to include all such variants into one lexeme.

This scenario is manifested in the signs for GRAY, which we discuss in Section “Examples of actual networks of variants” below.

### The Shared Vertex Scenario

**FIGURE 10:**
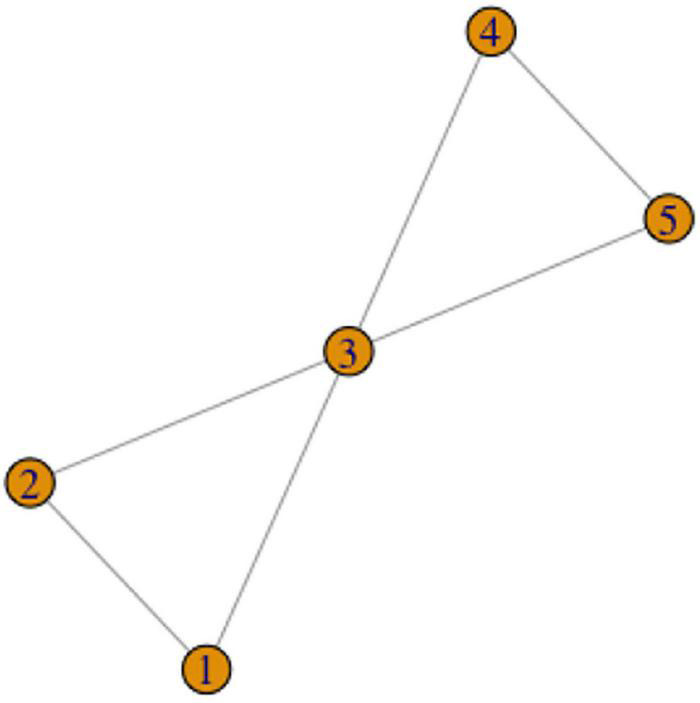
The Shared Vertex Scenario.

It is possible that two complex parts of the variant graph share a variant: two or more cycles of variants or a cycle and a chain can share one variant. For example, a concept X can have variants 1, 2, and 3, which form a cycle, and variants 3, 4, and 5, which also form a cycle (Figure 10).

A real-life example of this scenario is illustrated by the network of variants for the concept “pink” (see the Supplementary Materials for a full illustration). In fact, this concept is expressed by 11 variants, six of which are completely unrelated to the other variants, and the remaining five are related exactly in the way depicted in Figure 10.

As discussed in the previous section, intuitively it makes sense that all the variants within a cycle belong to the same lexeme. However, if two cycles share one variant, does this mean that they should both belong to the same lexeme? The answer to this question is less intuitive. On the one hand, the two connected cycles are overlapping in one vertex, and the shared vertex is connected to at least two variants in both cycles. If the variant shared between the two cycles is to be removed such that the graph turns into two separate components, these components can in some cases cease to remain to be cycles (e.g., if we remove the variant 3 in Figure 10). On the other hand, the connections within the cycles seem to be stronger than between the cycles.

### The Connected Component Scenario

**FIGURE 11:**
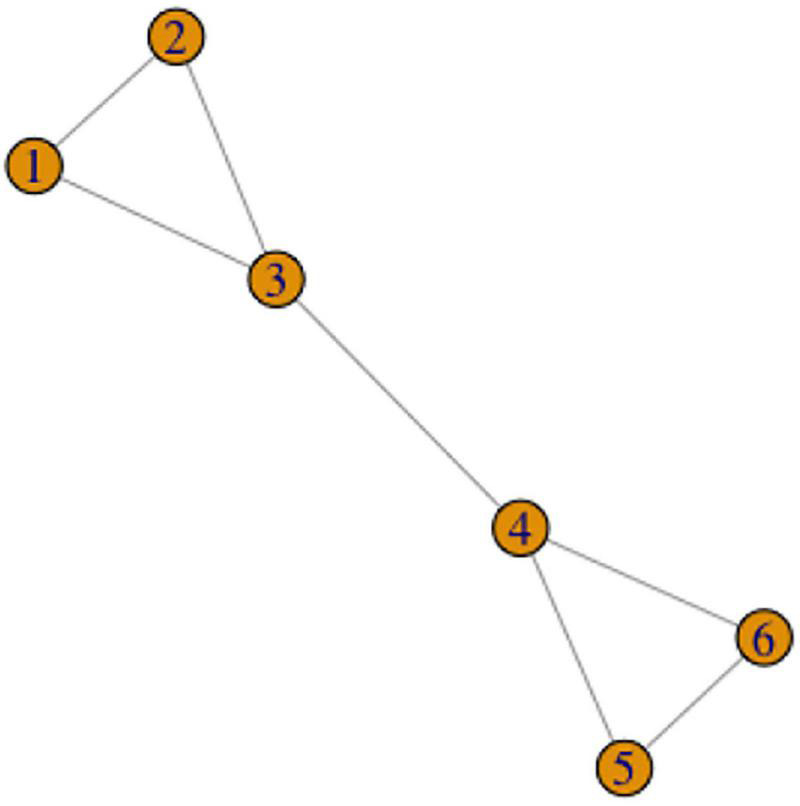
The Connected Component Scenario.

The final possibility is that multiple cycles and chains form a connected component of the variant graph, but without vertices shared by several cycles. The simplest example is represented in Figure 11: there are two fully connected cycles (123 and 456), and one variant in each cycle is connected to one variant in the other cycle (3 and 4). Basically, any connected component in graph theoretical terms (any part of the graph where there is a path from each variant to each other variant) falls under this scenario. A real-life illustration of this scenario is the variants of ORANGE in RSL, which will be discussed in the next section.

Note that the four scenarios from the previous section can all also be classified as the Connected Component Scenario – they are all examples of connected components with some additional restrictions. The Connected Component Scenario is thus the loosest scenario in terms of connections between variants, only requiring that all the variants are connected somehow – through some other variants. Furthermore, in this scenario, removal of some variants or connections between variants might not change the occurrence of cycles and chains within the remaining components. For instance, if in Figure 11 the variants 3 and 4 turn out to be phonologically unrelated, the cycles 123 and 456 would still remain. This shows that the more connected subparts of the graph are less dependent upon the presence of all the vertices and edges than in the Shared Vertex Scenario.

Thus, unless we decide that, in all four scenarios above, we should analyze all the variants as belonging to the same lexeme, we could not argue the same for this scenario either. Intuitively, the fact that two variants belong to a connected component might not be enough to classify them as belonging to the same lexeme. On the other hand, there is still a clear difference between variants belonging to the same component, and variants that do not belong to the same component, and thus are completely unconnected.

### Examples of Actual Networks of Variants

One example of actual networks of variants has been presented above: the variant signs for FATHER represented in Figures 7, 8 are all the variants for this concept found in our database. Thus, this is an example of a concept that has a cycle of variants, and no variants that are completely isolated. The same system of variants is also present for the concept MOTHER, where the only difference with the signs for FATHER is that the hand moves horizontally, between the right and left sides of the face (or vice versa).

**FIGURE 12:**
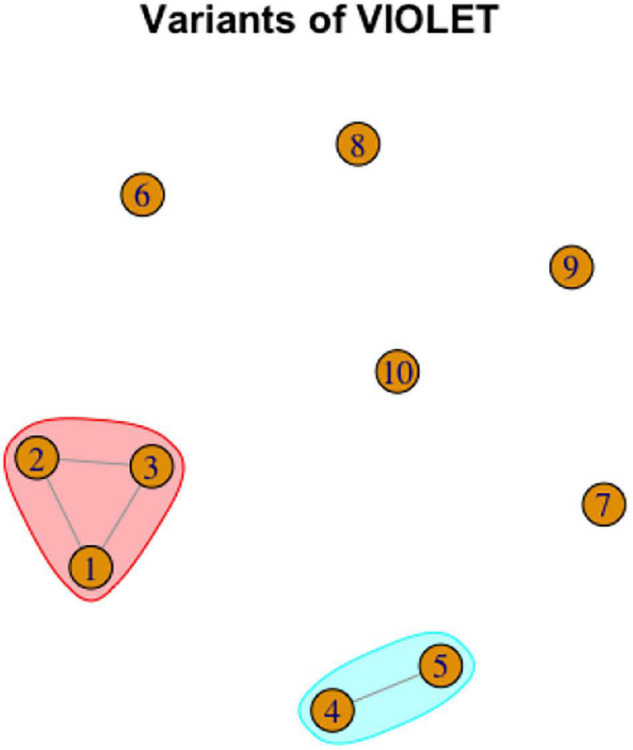
Graph representation of variation in VIOLET. Color shading represents lexemes.

Looking at color terms in RSL, we can observe large and interesting variation in the network complexity (the number of vertices and edges involved). For instance, RED has only two variants, which are only different in the handshape used, and thus they represent a single lexeme without complications. An example of the concept with a simple network of variants (but quite a large number of variants) is VIOLET (Figure 12).

As Figure 12 shows, variants 1, 2, and 3 are all phonologically related to each other, and thus they can be analyzed as a single lexeme. Variants 4 and 5 are phonologically related, so they represent another single lexeme. Variants 6 to 10 are unrelated to the other variants, so each is a lexeme.

However, most of the color terms manifest complex networks of variants, and illustrate the problematic scenarios identified above. This is the case for the concepts “white,” “black,” “yellow,” “pink,” “dark blue,” “light blue,” “gray” and “orange.”

**FIGURE 13:**
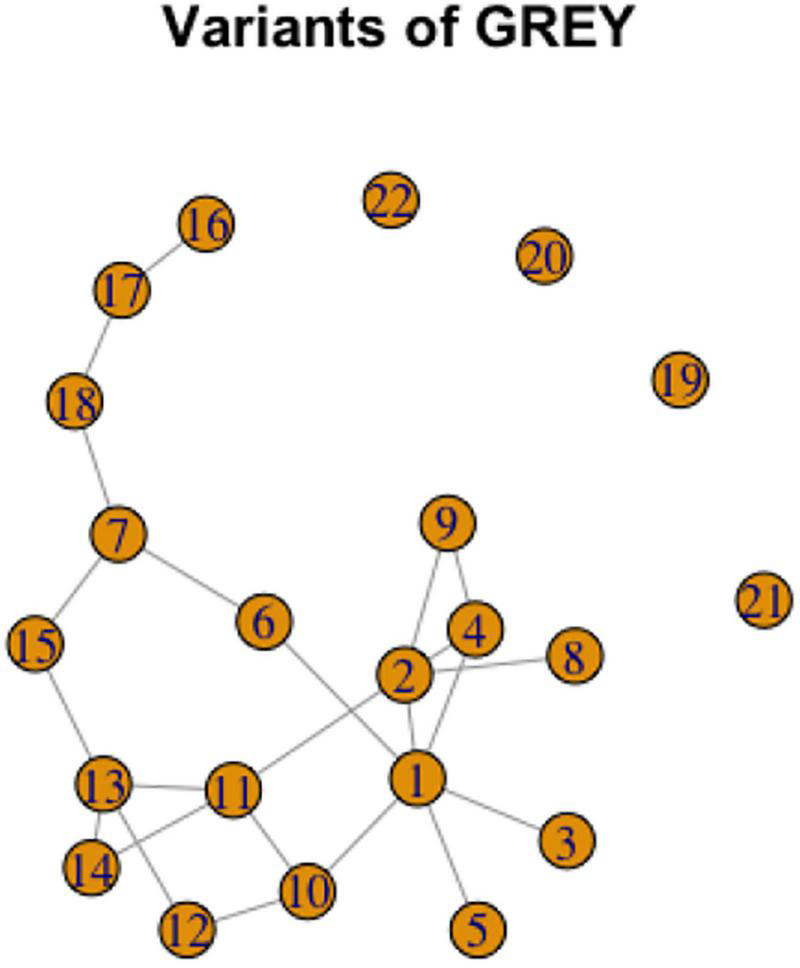
Graph representation of variation in GRAY.

To illustrate possible complexity, consider a graph representation for GRAY, Figure 13. This case illustrates the Overlapping Cycles Scenario. Specifically, variants 1,2,4,9,10,11,12,13,14 can be analyzed as a cycle; however, variants 6,7, and 15 are also all connected to this cycle, but in such a way that they do not form a single cycle together. In addition, there are variants 5, 3, and 8 each connected to one other variant in the same component, and variants 7, 18, 17, and 16 form a chain, thus illustrating the Chain Scenario. Finally, there are variants 19 to 22 which are unrelated to the other variants.

**FIGURE 14:**
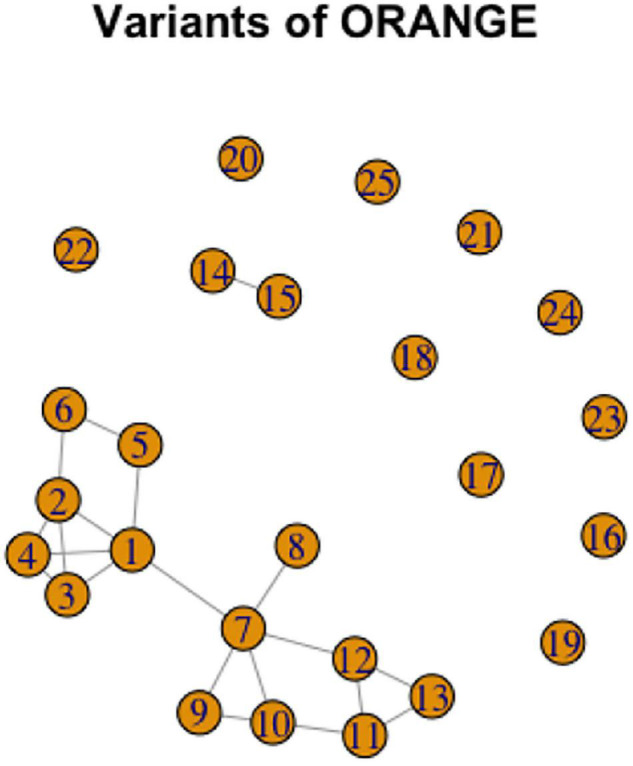
Graph representation of variation in ORANGE.

For another example of complexity, consider a graph representation of the variant network for ORANGE, Figure 14. This figure illustrates three of the five scenarios discussed above (for the Shared Vertex Scenario, see the representation for PINK in the Supplementary Materials for illustration).

ORANGE has 10 variants unrelated to the others, and thus comprising 10 separate lexemes. In addition, variants 14 and 15 together are phonologically related and thus form a lexeme. Looking at the connected part of the graph, we can see two cycles. The first one, represented by variants 1–6 is almost completely interconnected. The second cycle consists of variants 7,9,10,11,12. In addition, the two cycles are connected via the relation between variants 1 and 7 (the Connected Component Scenario). Abstracting away from the cycles, variants 1, 7, and 8 can be analyzed as a chain.

It is interesting to observe the pattern of variation here by looking at the specific forms of the signs. Variants 1 to 6 are all conducted in the neutral space, and while they vary in handshape and movement, they are interconnected enough to form a cycle. Variants 7 to 12 (excluding 8) are all conducted near the cheek, and also vary in handshape and movement. The two large groups have a connection via variants 1 and 7 which share the handshape and movement, but not the location. Thus, intuitively, each variant in each of the cycles belongs to a separate lexeme, but there is also a connection between the two lexemes that has to be acknowledged.

In the Supplementary Materials for this article, one can find graphical representations for all the color terms in the database analyzed so far and the video recordings for each variant of each concept.

## Discussion

The variability of the variant networks presented in the previous section makes it clear that the problem of distinguishing separate lexemes in such networks is non-trivial. It is definitely possible to develop a system of rules that will unambiguously identify lexemes even in the complex cases (Section ‘‘A possible system of rules’’)^8^. However, with such a system of rules in place, we still need to ask whether the binary distinction between lexical and phonological variants is really something to strive for.

### A Possible System of Rules

Starting with the Cycle Scenario, as discussed above, we have the intuition that all variants that are part of a cycle, should belong to a single lexeme. Recall the case of FATHER (Figures 7, 8). The signs for “father” vary along two dimensions (handshape and direction of movement), and all possible combinations of the three handshapes and two directions of movement are possible. It is possible to arbitrarily choose one of those dimensions as primary, and say, e.g., that FATHER consists of three lexemes (same handshape within each lexeme), or of two lexemes (same movement direction within each lexeme). However, first, we see no reasonable way of choosing one of the dimensions over the other. To sum up, we suggest the following rule:

(2) **The Cycle Rule:** Let X be the set of variants {1, 2, 3, etc.} for a concept. Let us represent the phonological relations between the variants as an undirected graph where the variants are vertices, and the variants that are distinguished by a single phonological parameter are connected by edges. If some subset of variants forms a cycle, we consider all of these variants belonging to the same lexeme.

We suggest that the Overlapping Cycles Scenario (Figure 9) can be analyzed in the same way by attributing all the variants in the overlapping cycles to one lexeme. This can be achieved by the following addition to the Cycle Rule in (3):

(3) **The Overlapping Cycles Rule:** Any variant that is connected to at least two variants within a cycle of variants belong to the same lexeme as the variants within the cycle.

We now move on to the Chain Scenario (Figures 4, 5). In this scenario, variants 1 and 2 are related, and so are 2 and 3, but not 1 and 3. We need to decide how many lexemes are manifested here, and which variants belong to which lexemes.

First, let’s handle the question of the number of lexemes^9^. For a chain of three variants 1, 2, and 3, we can propose one, two, or three lexemes. Proposing three lexemes is equivalent to removing the difference between lexemes and phonological variants completely, so we do not pursue this option further. Proposing that all the variants belong to the same lexeme might be acceptable, but this means violating the main criterion in (1), because now variants 1 and 3 belonging to the same lexeme are distinguished by more than one parameter. This leaves the option of the three variants belonging to two separate lexemes.

We propose that it is in fact possible to analyze a chain of three variants as two separate lexemes, and at the same time obey the main criterion, if we allow for one assumption: a single variant can belong to two separate lexemes. This means that in chains of variants, if variant 1 is related to 2, and variant 2 is related to 3, then 2 belongs to a lexeme together with 1, and 2 belongs to a lexeme together with 3. We then have two lexemes: Lexeme-A (1,2) and Lexeme-B (2,3).

In the case of the three variants of DARK.BLUE above (Figures 4, 5), this means the following: there are two lexemes, DARK.BLUE-A and DARK.BLUE-B. The first lexeme has two phonological variants: DARK.BLUE-11 and DARK.BLUE-12. The second lexeme also has two phonological variants: DARK.BLUE-12 and DARK.BLUE-13. We thus solve the question of where variant DARK.BLUE-12 belongs by stipulating that it belongs to two separate lexemes.

This solution might seem counterintuitive, but we argue that it is the best solution for the problem. First, in absence of other clues, it is usually impossible to decide where the “middle” variant in a chain should belong, as it is phonologically related to the two other signs to an equal extent. Second, having the same form belonging to different lexemes is a mechanism that is required in other scenarios anyway.

Consider homonymy: if we have concepts X and Y which are not related to each other, but both are expressed by the same form, we would say that this form indeed belongs to two different lexemes separately. To make an even more relevant example, imagine that we have two unrelated concepts X and Y, and both have two phonological variants. X can be pronounced as 1 or 2, and Y can be pronounced as 3 or 4. It so happens that the shapes of 2 and 4 are identical, but because the meanings they express are different, they are simply homonymous variants of different lexemes. Finally, the idea that the same form can belong to different lexemes has been applied in analyzing near synonyms in sign languages by Fenlon et al. (2015: 28–30), so our proposal only extends this idea to full synonyms (= lexical variants).

Note, however, that this solution does have a practical drawback in applied research. If we allow variant 12 to belong to two separate lexemes (DARK.BLUE-A and DARK.BLUE-B) in a lexical database, there will be two separate entries for the two lexemes, but they should both contain variant 12 as a phonological variant, which is cumbersome. Even more problematic is the fact that, if we then gloss a text where DARK.BLUE-12 occurs, there will be no way of deciding which of the two lexemes it should be identified as. A technical solution would be to use a double label (DARK.BLUE-A/DARK.BLUE-B), but again, this is not ideal. However, at the current moment, we do not see a better solution for the Chain Scenario.

With these solutions for the Cycle and Chain Scenarios, we also get the solutions for the Shared Vertex and the Connected Component Scenarios. For the former case (Figure 10), where a vertex is shared between two cycles, we can now easily say that the two cycles are two lexemes, and that the shared variant belongs to both lexemes.

For the Connected Component Scenario (Figure 11), again, each cycle within a connected component is analyzed as a separate lexeme, and if there is a chain, it is analyzed as consisting of several lexemes by the rules above, where variants can be shared between various chains and cycles.

**FIGURE 15:**
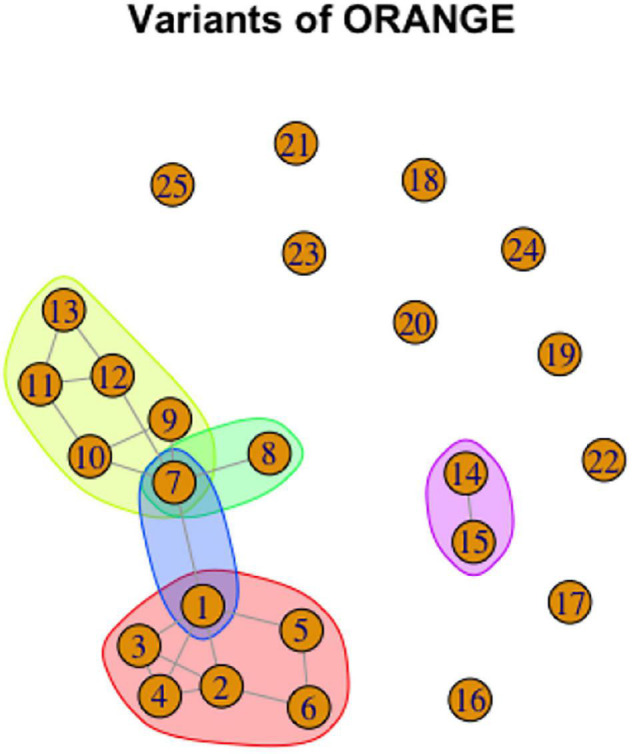
Graph representation of variation in ORANGE with lexemes indicated by color shading.

To give a specific example, consider again the graph representation of ORANGE, repeated here in a modified form as Figure 14. 10 variants are unrelated to the others, and thus manifest 10 separate lexemes. In addition, variants 14 and 15 together are phonologically related and thus form a lexeme. There are two cycles: variants 1–6 form a lexeme, and variants 7,9,10,11,12 form a lexeme. In addition, 1 and 7 together form a lexeme, and 7 and 8 together form a lexeme. The proposed lexemes are represented by color shading (for lexemes with more than one variant) in Figure 15.

### Problems With the Proposed System and Future Directions for Research

The system of rules proposed above has several problems, including technical and linguistic issues.

As discussed in the previous section, the decision to allow a single variant to belong to several lexemes introduces technical problems for lemmatization for dictionaries (Hochgesang et al., 2018), but especially for lemma-based glossing of corpus data (Mesch and Wallin, 2015). For such variants that are attributed to multiple lexemes, technical solutions exist. One solution already mentioned above is to use glosses in which both (or all) lexemes that share this variant are named. Another solution is to arbitrarily assign the shared variant to one of the lexemes, which is not problematic as long as this assignment is clearly registered in a protocol.

Linguistic issues are more serious, in our opinion. The proposed system of rules is built in order to preserve the binary opposition between separate lexemes and variants within a single lexeme. However, as we have seen, empirically the variety of relations between variants is more rich. Specifically, we observe the following cases of relations between pairs of variants:

(1) Two variants can have no relation to each other, not even through other variants (e.g., 20 and 21 on Figure 14). Clearly, such variants belong to separate lexemes.
(2) Two variants are not directly phonologically related, but there are some intermediary variants such that one can form a path (chain) of phonological relations between the two variants (e.g., 6 and 8 on Figure 14).
(3) Two variants are not directly phonologically related, but they form a part of a connected network of variants (they are part of the same cycle or overlapping cycles, e.g., 10 and 13 on Figure 14).
(4) Two variants are directly phonologically related (e.g., 1 and 2 on Figure 14).

Based on existing research, cases (1) and (4) above are clear. In (1), the pairs of variants clearly belong to two separate lexemes. In (4), the pairs of variants clearly belong to the same lexeme. The intermediary cases (2) and (3), however, need to be acknowledged, and probably analyzed separately in linguistic and psycholinguistic research. It might be the case that instead of having a binary opposition of lexemes vs. phonological variants, we need to have at least four categories (corresponding to the list above): **separate lexemes, connected variants, variants within cycles/overlapping cycles, phonological variants**.

In order to test the validity of these categories, we suggest the following steps:

• Analysis of networks of variants similar to the one presented in this paper should be applied to other signs in RSL and to other sign languages in order to explore and discover the possible configurations. More complex scenarios can be discovered that we have not yet identified. It might be possible to use existing databases of lexical variation for such research, for instance, the database for Chinese Sign Language (Chen and Gong, 2020) would be very suitable for this type of analysis.
• It would be interesting to explore how various phonological processes (van der Hulst and van der Kooij, 2021) affect the configurations of variant networks^10^. For example, a weak hand drop, if phonologized, might lead to an emergence of a phonological new variant, and this variant can be incorporated in already complex network of existing variants and disturb or modify its structure. Both theoretical and actual scenarios should be explored.
• Sociolinguistic properties of the different categories should be explored. For example, it might be the case that the choice between separate lexemes and connected variants are explained by a factor which does not explain the choice of variants within a cycle or phonological variants, etc. In other words, it should be tested whether the four categories are distinguished in actual use.
• Psycholinguistic experiments should be carried out in order to explore whether the different categories we identify are distinguished in production and perception by native signers.

Furthermore, it can be useful to try and enrich the graph representation of networks by incorporating other factors:

• Frequency of variants should also be analyzed if possible. For example, it might turn out that some variants are much less frequent than others that they are phonologically related to (see, e.g., Chen and Gong for such findings for Chinese Sign Language); this information might be used to enrich the graph representation of the networks, and provide insights into the typology of configurations.
• Representations can be further enriched with phonological information. For example, one can add some indication of what the common components are between the variants that are phonologically related in order to study whether different components typically occur in different types of graphs (e.g., in cycles vs. chains).

Finally, it might be worth testing community detection algorithms on these variant networks (Fortunato, 2010). It would be interesting to see whether automatically detectable communities correspond to lexemes in our definition and to intuitions of native signers, at least for larger networks. As we have mentioned above, community detection algorithms may not be effective in detecting lexemes in such small networks, but some specific community detection methods offer interesting approaches that may allow us to explore structures of variant networks as weighted graphs using phonological information to calculate a numerically specific degree of overlap between different variants.

## Summary

In this paper, we demonstrated the patterns of lexical variation in sign languages in terms of phonological relatedness between the variants. In order to do so, we analyzed kinship terms and color terms in a newly created database of lexical variation in RSL. We proposed the use of a graph representation as a tool of visualizing relationships between variants.

We discussed that the usual approach to distinguishing phonological and lexical variants of signs does not work in some cases (the problematic scenarios). These scenarios turn out to be well attested in RSL. Further, by studying these configurations, we therefore developed a system of rules to handle such cases. While the system is somewhat complicated, it allows to fully handle the extent of the variation at play.

At the same time, we conclude that the actual patterns of possible relations between variants has to be acknowledged. Instead of focusing on the binary distinction between lexemes and phonological variants, it might be necessary to distinguish at least four categories: separate lexemes, connected variants, variants within a cycle, phonological variants. However, further linguistic and psycholinguistic research is necessary to establish psychologic reality of these categories.

## Data Availability Statement

The datasets presented in this study can be found in online repositories. The names of the repository/repositories and accession number(s) can be found in the article/Supplementary Material.

## Ethics Statement

Ethical review and approval was not required for the study on human participants in accordance with the local legislation and institutional requirements. The participants provided their written informed consent to participate in this study. Written informed consent was obtained from the individual(s) for the publication of any potentially identifiable images or data included in this article.

## Author Contributions

VK wrote the manuscript with contributions from the other authors. All authors participated in designing the study, developing data collection, annotation of the database, and analyzing the data.

## Conflict of Interest

The authors declare that the research was conducted in the absence of any commercial or financial relationships that could be construed as a potential conflict of interest.

## Publisher’s Note

All claims expressed in this article are solely those of the authors and do not necessarily represent those of their affiliated organizations, or those of the publisher, the editors and the reviewers. Any product that may be evaluated in this article, or claim that may be made by its manufacturer, is not guaranteed or endorsed by the publisher.

## Footnotes

1 For consistency, we use numbers (1, 2, 3, …) to represent variants (signs with distinct shapes) and letters (A, B, C, …) to represent lexemes (which can consist of multiple variants).

2 In fact, Chen and Gong (2020: 7) decided to analyze signs which overlap in at least **two** parameters as phonologically related in their study of variation in Chinese Sign Language.

3 The website’s interface is only in Russian at the moment of publication (December 2021), but the English interface is planned to be added in near future.

4
https://rsl-research.garagemca.org/

5 It is, however, unlikely that such a process would apply for our data set, because the signs were produced without any context, and with the same stimuli for all the signers.

6 Two of the authors are hearing CODA signers of RSL, all of the authors are fluent RSL signers.

7 More than two variants can also be mutually adjacent, forming what is known as a clique.

8 After identifying lexeme boundaries, a separate step necessary in dictionary creation is determining the citation form, when factors such as frequency and iconicity of the candidate variants are taken into account (Cormier et al., 2012). We do not further discuss this aspect, focusing on the initial step of determining the number of lexemes and attributing the variants to these lexemes.

9 This analysis is inspired by treatment of dialectal chains proposed by Hammarström (2008) with regards to determining the number of languages in situations of dialectal chains.

10 We thank a reviewer for this suggestion.
