# Supplementary material for: Exploring Networks of Lexical Variation in Russian Sign Language
Source: Front Psychol. 2022 Jan 5;12:740734. doi: 10.3389/fpsyg.2021.740734 (PMC8766300; doi:10.3389/fpsyg.2021.740734)

Supplementary Material

# Explanation

The Supplementary Material for the article “Exploring networks of lexical variation in Russian Sign Language” contain Figures (graphs) for the variant networks of the colour terms analysed. The video recordings of each of the variant can be found here: <https://osf.io/7h3f6/>.

In the Figures, connected vertices represent phonologically related variants. Colour shading is used for proposed lexemes where a lexeme contains more than one variant, following the rule system described in the paper. The numbers in the graph correspond to the numbers in the video titles (e.g. the video DARK.BLUE-1 illustrates variant 1 in the graph). Note that some videos contain multiple variants, mentioned in the filenames.

The colour RED is not represented, because it is expressed by only two phonologically related variants.

# Supplementary Figures

#
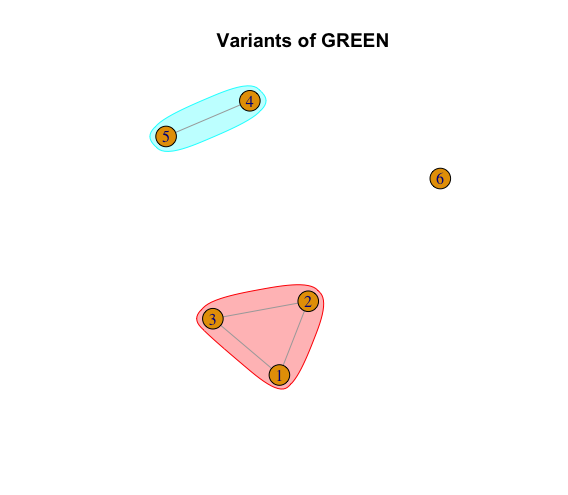

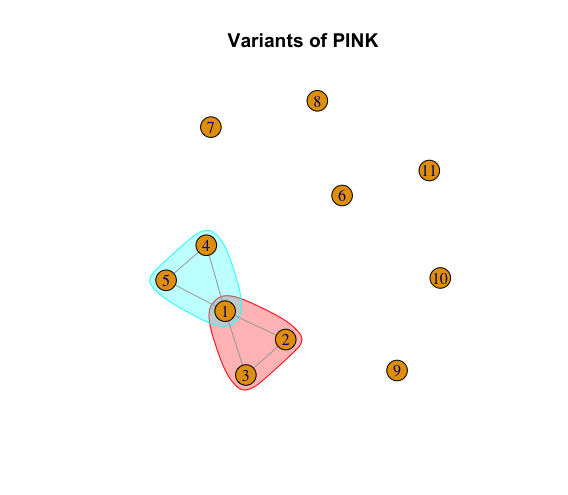

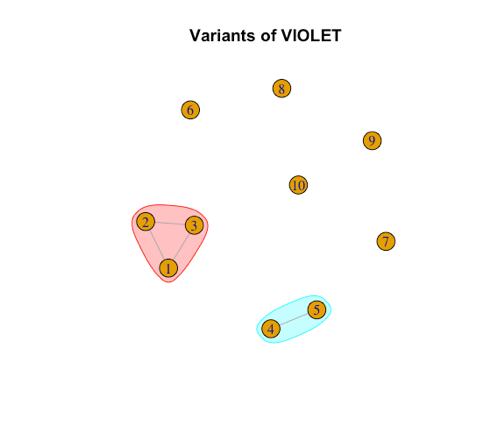

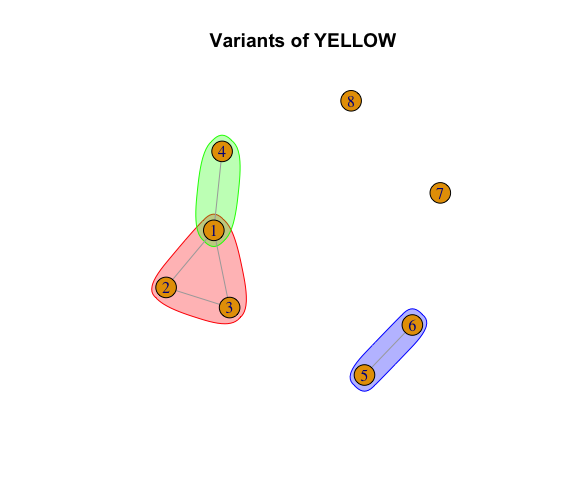

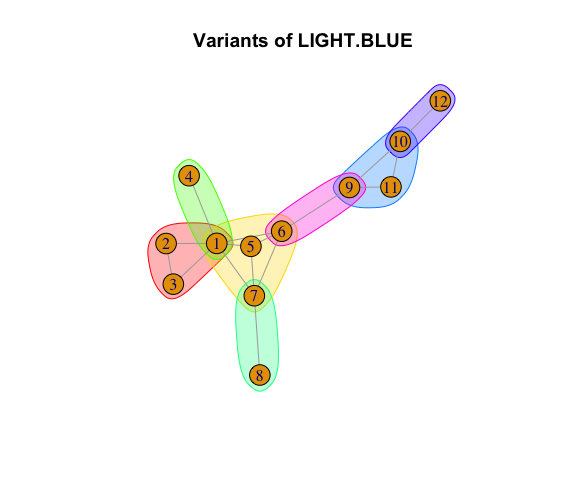

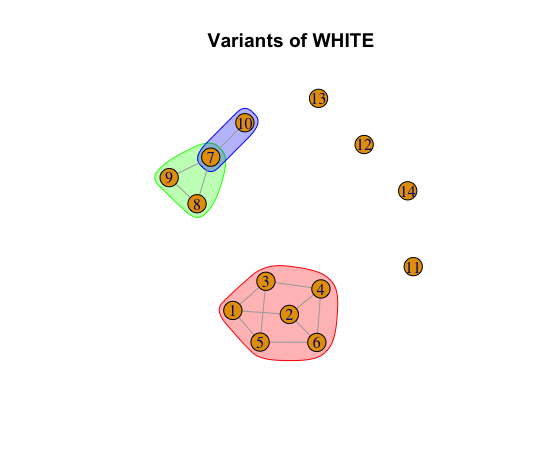


#
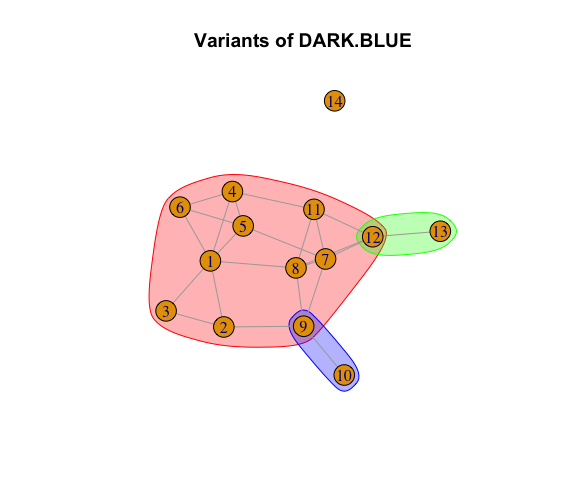

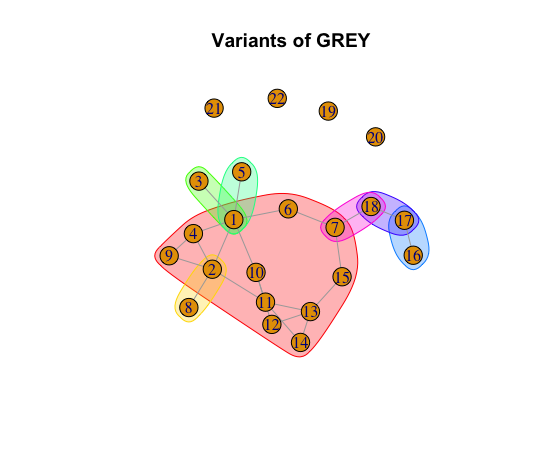


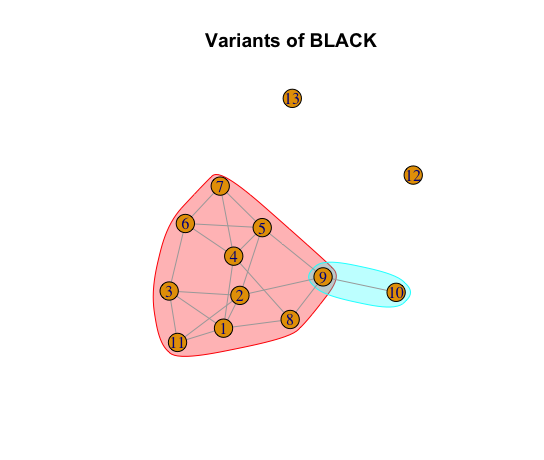

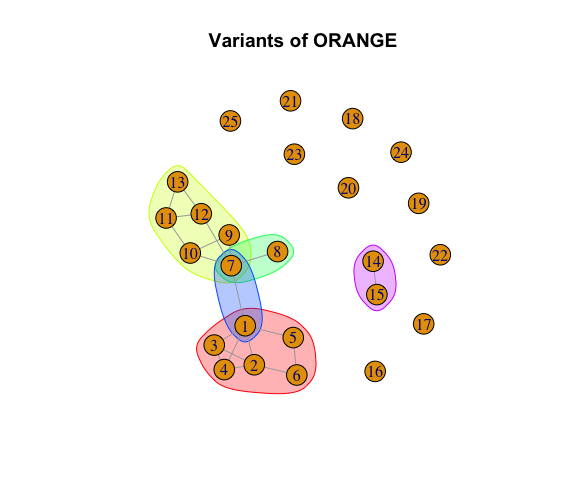

Supplement: Supplementary file 1 [file Table_1.DOCX]
